# Novel Insights Into Tissue-Specific Biochemical Alterations in Pediatric Eosinophilic Esophagitis Using Raman Spectroscopy

**DOI:** 10.14309/ctg.0000000000000195

**Published:** 2020-07-21

**Authors:** Girish Hiremath, Andrea Locke, Giju Thomas, Rekha Gautam, Sari Acra, Hernan Correa, Evan S. Dellon, Anita Mahadevan-Jansen

**Affiliations:** 1 Division of Pediatric Gastroenterology, Hepatology, and Nutrition, Monroe Carell Jr. Children's Hospital at Vanderbilt, Vanderbilt University Medical Center, Nashville, Tennessee, USA;; 2 Vanderbilt Biophotonics Center, Department of Biomedical Engineering, Vanderbilt University, Tennessee, USA;; 3 Division of Pediatric Pathology, Monroe Carell Jr. Children's Hospital at Vanderbilt, Vanderbilt University Medical Center, Nashville, Tennessee, USA;; 4 Division of Gastroenterology and Hepatology, University of North Carolina at Chapel Hill, North Carolina, USA.

## Abstract

**INTRODUCTION:**

Elucidating esophageal biochemical composition in eosinophilic esophagitis (EoE) can offer novel insights into its pathogenesis, which remains unclear. Using Raman spectroscopy, we profiled and compared the biochemical composition of esophageal samples obtained from children with active (aEoE) and inactive EoE (iEoE) with non-EoE controls, examined the relationship between spectral markers and validated EoE activity indices.

**METHODS:**

*In vitro* Raman spectra from children with aEoE (n = 8; spectra = 51) and iEoE (n = 6; spectra = 48) and from non-EoE controls (n = 10; spectra = 75) were acquired. Mann-Whitney test was used to assess the differences in their Raman intensities (median [interquartile range]) and identify spectral markers. Spearman correlation was used to evaluate the relationship between spectral markers and endoscopic and histologic activity indices.

**RESULTS:**

Raman peaks attributable to glycogen content (936/1,449 cm^−1^) was lower in children with aEoE (0.20 [0.18–0.21]) compared with that in non-EoE controls (0.24 [0.23–0.29]). Raman intensity of proteins (1,660/1,209 cm^−1^) was higher in children with aEoE compared with that in non-EoE controls (3.20 [3.07–3.50] vs 2.91 [2.59–3.05]; *P* = 0.01), whereas that of lipids (1,301/1,260 cm^−1^) was higher in children with iEoE (1.56 [1.49–1.63]) compared with children with aEoE (1.40 [1.30–1.48]; *P* = 0.02). Raman peaks attributable to glycogen and lipid inversely correlated with eosinophilic inflammation and basal zone hyperplasia. Raman mapping substantiated our findings.

**DISCUSSION:**

This is the first study to identify spectral traits of the esophageal samples related to EoE activity and tissue pathology and to profile tissue-level biochemical composition associated with pediatric EoE. Future research to determine the role of these biochemical alterations in development and clinical course of EoE can advance our understanding of EoE pathobiology.

## BACKGROUND

Eosinophilic esophagitis (EoE) is an immune-mediated disease characterized clinically by symptoms of esophageal dysfunction and histologically by an intense eosinophilic inflammation involving the esophageal mucosa (1). In the United States, it is estimated to affect 1 in 2,000 individuals, across all ages (2). Genetic, epigenetic, and environmental studies have revealed a strong hereditability pattern, involvement of multiple gene polymorphisms including in thymic stromal lymphopoietin, calpain 14, and STAT6, and have demonstrated that dietary and environmental triggers are closely linked with the development of EoE (3). Despite these notable advances, our understanding of the pathogenesis of EoE remains incomplete. This is partly due to our limited insight into the biomolecular and biochemical alterations associated with transformation of a normal (or unaffected) esophageal tissue to a diseased tissue affected by EoE as the biochemical changes are more closely related to the tissue pathology or the disease phenotype than the transcriptome or the proteome (4).

Multiple light-based approaches are being developed to elucidate cellular, subcellular, and structural changes in EoE (5–8). However, these are not suitable for unraveling biochemical composition and spatial distribution of biologically active molecules. Raman spectroscopy is a validated optical technique suitable for profiling biochemical composition of the tissue, *in vitro* and *in vivo*, without requiring any staining or dyes (also known as label-free profiling) (9,10). When monochromatic laser light interacts with the chemical bonds of a molecule in a specimen, it results in exchange of energy (either a gain or loss) with the vibrational modes of these chemical bonds. These changes can be detected and processed as Raman spectra. The spectra are plotted as a function of shifts in the wavenumber (i.e., inverse wavelength) about the incident monochromatic light. These wavenumber shifts are unique for individual molecules (such as glycogen, proteins, lipids, nucleic acids, amino acids, and collagen) owing to their conformation, structure, and/or environment and reflect the biochemical fingerprint (e.g., amide band of protein and phosphate stretching in DNA backbone) for that particular molecule (11,12). A reference table of these peak assignments has been compiled and is widely accepted in the Raman community (13). This versatile technique has been used to interrogate discrete chemical information at distinct positions within the sample (spectral analysis) and to delineate biochemical information coupled with its spatial distribution (Raman mapping) (14) for precancerous and cancers conditions, including those involving the esophagus, in animal models and in humans (15,16).

To date, Raman spectroscopy has not been applied to EoE outside of a murine model (17) and never to human tissue. Our study addresses this important knowledge gap in the field. The primary aim of this study was to use Raman spectroscopy to profile and compare the *in vitro* biochemical composition of the esophageal samples collected from children with and without EoE and to identify candidate spectral markers associated with EoE. The secondary aims were to investigate the relationship between candidate spectral markers and both EoE activity status and validated EoE activity indices. We hypothesized that Raman spectroscopy could provide spectral traits that are linked to EoE because of the underlying biochemical changes distinctly associated with the pathogenetic process.

## METHODS

### Study subjects and groups

This was a prospective study of children (6–18 years) undergoing esophagogastroduodenoscopy (EGD) with biopsies at the Monroe Carell Jr. Children's Hospital at Vanderbilt for assessment of their EoE status or for investigating upper gastrointestinal symptoms suggestive of EoE. Patients with EoE were those newly or previously diagnosed with EoE as defined per the 2011 Consensus recommendations (18). Specifically, affected subjects at the time of original diagnosis were required to have symptoms of esophageal dysfunction and a peak eosinophil count (PEC) of ≥15 eosinophils per high-power field (eos/hpf) in at least one of the multiple esophageal biopsies after 8 weeks of proton pump inhibitor therapy and in the absence of other causes of esophageal eosinophilia. Children were classified as active EoE (aEoE) if their biopsies revealed a PEC of ≥15 eos/hpf or as inactive EoE (iEoE) if they had a PEC of <15 eos/hpf. Individuals without a previous diagnosis of EoE and undergoing EGD for upper gastrointestinal symptoms (such as abdominal pain, nausea, and vomiting) and whose biopsies revealed a PEC <15 eos/hpf were considered as non-EoE controls. This included children with gastroesophageal reflux disease. Subjects with other forms of eosinophilic intestinal disorders (e.g., eosinophilic gastroenteritis), inflammatory bowel disease, connective tissue disorder, esophageal varices, and previous esophageal surgery were excluded. The study was approved by Vanderbilt University Institutional Review Board (protocol nos.: 151341 and 160785), and informed consent/assent was obtained from all subjects/parents/caregivers as appropriate.

### Clinical information

Demographic data (including age, sex, and ethnicity), clinical data (such as presence of nausea, vomiting, reflux, dysphagia, and abdominal pain), allergic comorbidities (with allergic rhinitis, eczema, and asthma), and medication exposure (as in antihistamines, nasal topical steroids, proton pump inhibitor, and topical corticosteroids) were gathered from the electronic medical records.

### Endoscopic findings

During the EGD, esophageal endoscopic signs were scored per the validated EoE endoscopic reference score (EREFS) for edema (0–2), rings (0–3), exudates (0–2), furrows (0–2), and strictures (0–1). Minor features such as narrowing of the esophagus and crepe paper esophagus were also evaluated (19). Features such as edema, exudates, and furrows represented an inflammatory phenotype, and rings and strictures indicated a fibrostenotic phenotype. Total EREFS score was calculated by combining individual values (range 0–10), with higher scores representing more severe endoscopic findings. All endoscopies and EREFS scoring were performed by a single investigator (G.H.) who had access to clinical information but was blinded to histopathological and Raman data.

### Esophageal biopsies

We obtained 3–4 esophageal mucosal biopsies at both the proximal and the distal esophagus (total of 6–8 biopsies) per subject using a disposable EndoJaw forceps (Alligator Jaw Step Fenestrated with needle; Olympus Medical Systems, Japan) for clinical care purposes. In addition, we collected a single distal esophageal (within 5 cm from the lower esophageal sphincter) biopsy from a site adjacent to the distal clinical biopsies for Raman analysis. Each biopsy was approximately 2 mm^3^ in size. The biopsies for clinical care purposes were placed in formaldehyde solution and submitted for H&E staining and histologic assessment, and the sample collected for Raman analysis was flash frozen at the point of collection and stored at −80°C.

### Histologic evaluation

The biopsies for clinical care purposes were assessed for PEC (eos/hpf; hpf = 0.237 mm^2^) in the fragment with the highest number of eosinophils in the squamous epithelium. For the purposes of this study, the clinical biopsies were reevaluated to quantify the degree of abnormality (grade score) of the 8 pathologic features per the EoE histology scoring system (EoEHSS) (20) (see Supplementary Material, Supplementary Digital Content 7, http://links.lww.com/CTG/A306). All biopsies were examined and scored by single examiner (H.C.) who had access to demographic and clinical information but was masked to Raman data.

### Raman data

#### Instrumentation and acquisition of Raman spectra

*In vitro* Raman spectra were obtained using an inVia Raman Confocal microspectroscope (Wotton-Under-Edge, UK) (see Supplementary Material, Supplementary Digital Content 7, http://links.lww.com/CTG/A306). Each sample was first thawed at room temperature, washed 3 times with distilled water, and then placed on a stainless steel disc. The disc was then transferred to the inVia Raman Confocal microspectroscope under ×50 objective (numerical aperture = 0.75) with a 0.5-µm spot size and 2–5 μm depth penetration (21). Multiple Raman spectra, across a wavenumber range of 600–1,700 cm^−1^ were acquired from 6 to 8 discrete points from each sample with a 785-nm diode laser (Innovative Photonic Solution, NJ) excitation source (10 mW) using an exposure time of 10 s with 5 accumulations per spectra. Raman measurements were taken from both surfaces (mucosal and adventitial) to ensure representative spectra.

#### Raman mapping

Raman mapping was performed on an independent set of distal esophageal biopsies representing each of the study groups. A 20-μm section of the frozen samples were obtained and fixed on calcium fluoride slides. Adjacent 5-μm section was obtained for H&E analysis. Raman mapping was conducted under ×50 objective with ∼20 mW power at the sample. This allowed us to get optimal signal-to-noise ratio while minimizing any injury to the tissue architecture or its composition. A region of interest was selected for measurement using an XY translational stage, and the spectra were collected by scanning across the region of interest with a step size of 6 μm with a total acquisition time of 1 second per point and a total of 9–10 hours per sample. The images were then processed using cosmic ray removal, fluorescence background subtraction, and normalization to the calcium fluoride microscope slide background using 4.2 WiRE software (Renishaw plc, UK).

### Data processing and statistical analysis

Descriptive statistics including counts and percentages for categorical variables, and median (range) for continuous variables were used to characterize the cohort. A Bonferroni correction was used to adjust for multiple comparisons, and statistical significance was determined for a *P* value of ≤0.003.

The individual Raman spectra from each of the samples were obtained and processed in MATLAB (Natick, MA) by performing Savitzky–Golay smoothing to reduce noise, background fluorescence subtraction, and mean normalization (22). After this, the median (interquartile range) was calculated across the entire normalized Raman spectra and for each study group. Subsequently, discriminant spectral traits characteristic to each study groups were identified using the Mann-Whitney U test. Spearman correlation was used to determine the strength and direction of relationship between the candidate spectral markers and each of the components of EREFS and EoEHSS. The statistical significance was ascertained at *P* value ≤0.05. OriginPro (OriginLab, North Hampton, MA) was used for statistical analyses and illustration of data.

## RESULTS

### Raman spectral analysis

#### Subject characteristics

In all, esophageal samples from 24 children were analyzed (EoE; n = 14, non-EoE controls; n = 10). Of the 14 children with EoE, 8 (57%) had aEoE and 6 (43%) had iEoE. The median (interquartile range) age of the cohort was 13 (9–17) years, with 81% being boys and 79% being white. EREFS was higher for children with aEoE (median [range]: 2 [1–4]) compared with those of children with iEoE (0 [0–1]) and non-EoE controls (0 [0–0]). Most children with EoE had an inflammatory phenotype. The EoEHSS was higher for children with aEoE (0.31 [0–0.58]) when compared with those of children with iEoE (0.04 [0–0.13]) and non-EoE controls (0 [0–0.05]), with presence of basal zone hyperplasia (BZH) being higher in children with aEoE (87%) compared with those of children with iEoE (33%) and non-EoE controls (10%) (Table 1).

**Table 1.**
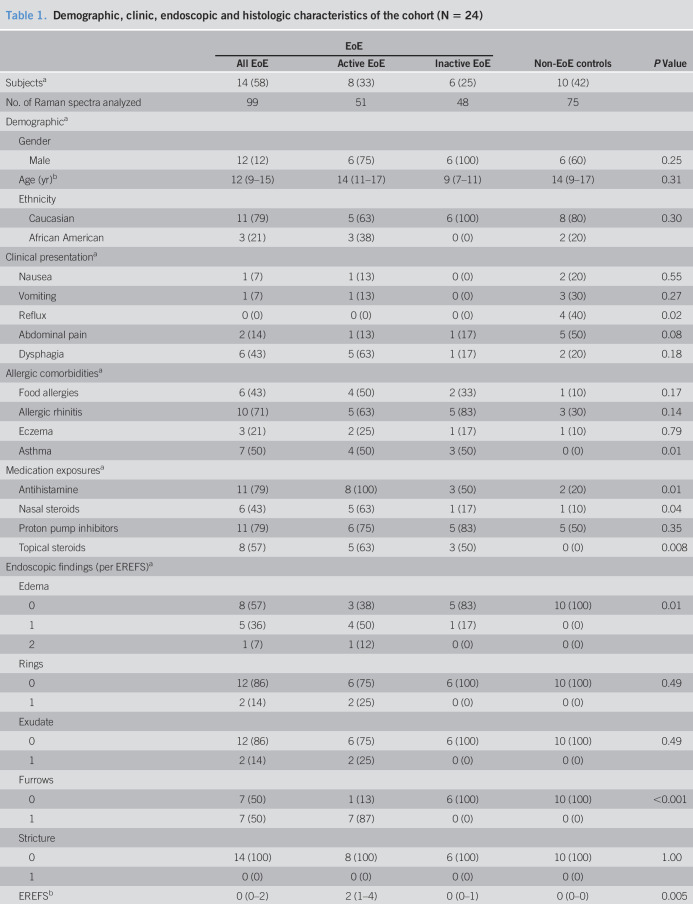

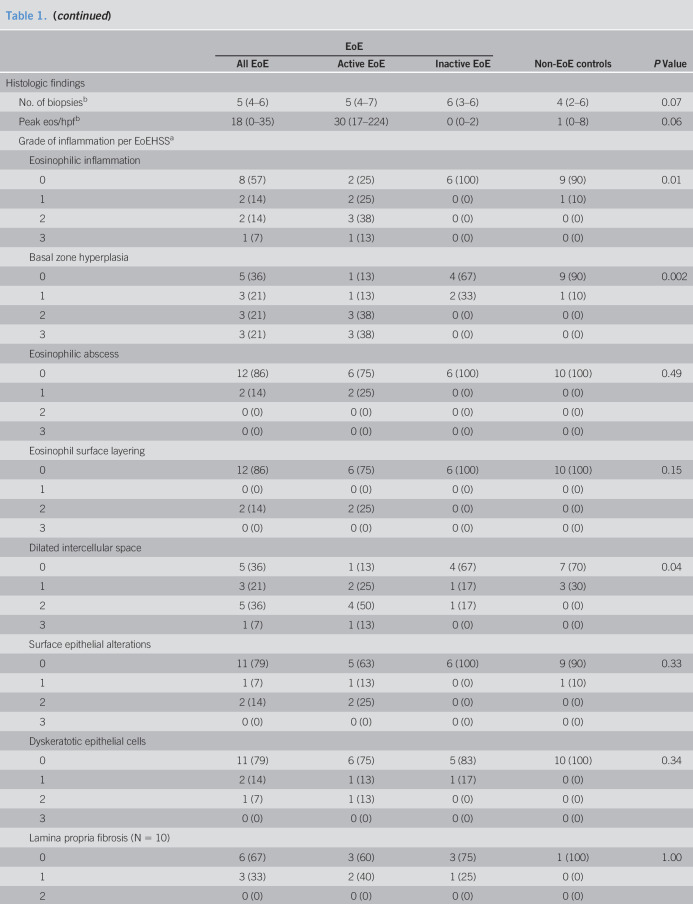

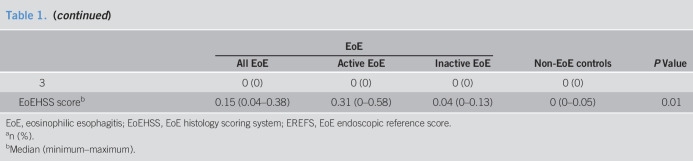
Demographic, clinic, endoscopic and histologic characteristics of the cohort (N = 24)

#### Panel of spectral markers distinguish children with EoE from non-EoE controls

In all, 174 spectra were analyzed. Of these, 99 were from cases with EoE and 75 from non-EoE controls (n = 10), which included samples from patients diagnosed with gastroesophageal reflux disease (n = 6). Of the EoE samples, 51 were acquired from cases with aEoE (n = 8) and 48 from cases with iEoE (n = 6).

**Figure 1.**
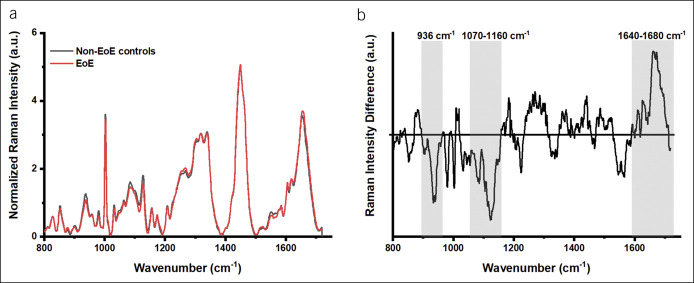
(**a**) Normalized *in vitro* Raman intensities of EoE and non-EoE controls. (**b**) The difference spectrum of the mean normalized Raman intensities between the EoE and non-EoE controls. EoE, eosinophilic esophagitis.

First, we characterized the spectral signature of EoE tissue irrespective of the activity status. When compared with samples from non-EoE controls, EoE samples had significant differences in the Raman spectra at ∼936 cm^−1^ (C–O stretch: glycogen, C–C alpha-helix proteins), 1,124 cm−^1^ (C–O stretch: glycogen), 1,070–1,160 cm^−1^ (C–C stretching mode of lipids, PO_2_^−^ stretching mode of DNA, C–O stretch of carbohydrates, and C–C and C–N stretching mode of proteins), 1,200–1,270 cm^−1^ (amide III: coupling of C–N stretching and N–H bending modes of proteins), 1,301 cm^−1^ (CH_2_ twist/wag/deformation of lipids), 1,440–1,450 cm^−1^ (CH_2_ bending modes of proteins and lipids), and 1,640–1,680 cm^−1^ (amide I, predominantly the C=O in proteins and lipids) (Figure 1). The ratiometric analysis of Raman bands of relating to glycogen content at 936/1,449 cm^−1^ (0.20 [0.19–0.22] vs 0.24 [0.23–0.29]; *P* = 0.01) and at 1,124/1,449 cm^−1^ (0.31 [0.28–0.32] vs 0.34 [0.32–0.39]; *P* = 0.01) was significantly lower in EoE samples when compared with samples from non-EoE controls. By contrary, the ratiometric analysis of Raman bands at 1,660/1,209 cm^−1^ attributable to protein content was significantly higher in EoE samples compared with those in samples from non-EoE controls (3.19 [3.06–3.48] vs 2.90 [2.55–3.04]; *P* = 0.01).

#### Candidate spectral markers associated with EoE status

**Figure 2.**
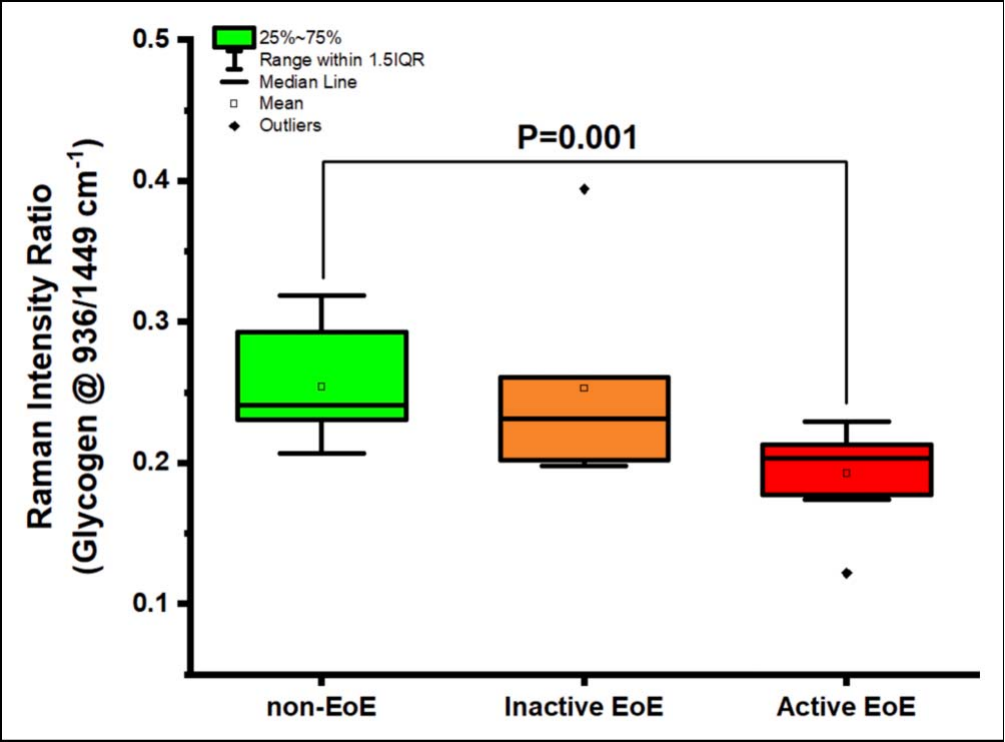
Comparing Glycogen content (Raman intensity ratio of 936/1,449 cm^−1^) between non-EoE controls, inactive EoE and active EoE samples. EoE, eosinophilic esophagitis; IQR, interquartile range.

**Figure 3.**
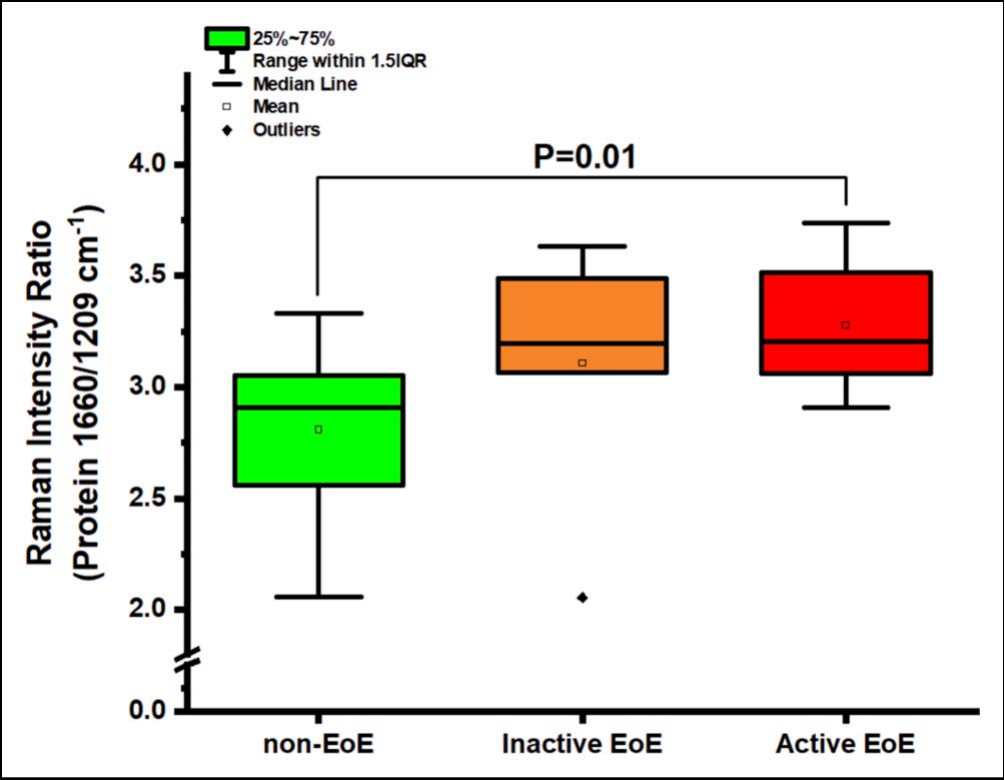
Comparing the abundance of protein (Raman intensity ratios at 1,660/1,209 cm^−1^) between non-EoE controls, inactive EoE and active EoE. EoE, eosinophilic esophagitis; IQR, interquartile range.

**Figure 4.**
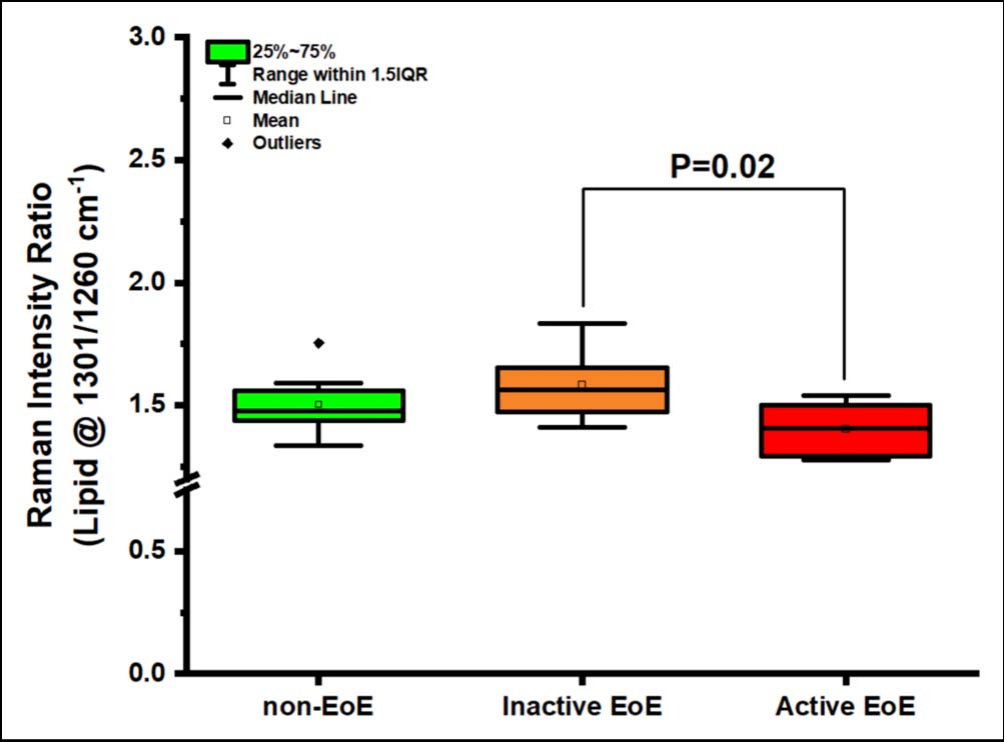
Differences in the content of lipids (at Raman intensity ratio of 1,301/1,260 cm^−1^) between non-EoE controls, inactive EoE, and active EoE. EoE, eosinophilic esophagitis; IQR, interquartile range.

Next, we compared spectral signatures between aEoE, iEoE, and non-EoE controls and investigated whether EoE activity status was associated with distinct spectral markers. Key differences were observed between aEoE and iEoE samples in the ∼936 cm^−1^, 1,003 cm^−1^, 1,440–1,445 cm^−1^, and 1,650–1,660 cm^−1^ regions (see Figure 1, Supplementary Digital Content 1, http://links.lww.com/CTG/A300). The ratio of 936/1,449 cm^−1^ (0.20 [0.17–0.21] vs 0.24 [0.23–0.29]; *P* = 0.001) and of 1,124/1,449 cm^−1^ (0.31 [0.28–0.32] vs 0.34 [0.32–0.39]; *P* = 0.01) assigned to glycogen content was significantly lower in aEoE samples when compared with that in samples of non-EoE controls (Figure 2, and see Figure 2, Supplementary Digital Content 2, http://links.lww.com/CTG/A301). The ratio of 1,660/1,209 cm^−1^, indicative of relative protein content, was also significantly higher in aEoE samples when compared with that in samples of non-EoE controls (3.20 [3.07–3.50] vs 2.91 [2.59–3.05]; *P* = 0.01) but comparable with that in iEoE samples (3.20 [3.07–3.50] vs 3.20 [3.09–3.42]; *P* = 0.85) (Figure 3). Similarly, the ratio of 1,450/1,084 cm^−1^, also attributable to protein content, was significantly higher in aEoE samples when compared with that in samples of non-EoE controls (3.53 [3.36–3.66] vs 3.22 [2.82–3.50]; *P* = 0.03) (see Figure 3, Supplementary Digital Content 3, http://links.lww.com/CTG/A302). The spectral ratio of 1,301/1,260 cm^−1^, related to lipid content, was higher in iEoE samples when compared with that in aEoE samples (1.56 [1.49–1.63] vs 1.40 [1.30–1.48]; *P* = 0.02) and comparable with that in samples of non-EoE controls (Figure 4). These findings suggest that spectral markers indicative of decreased glycogen content and increased protein content can allow differentiation between cases with aEoE and iEoE or non-EoE controls, and those representing increased lipid content can allow differentiation between cases with iEoE and aEoE or non-EoE controls.

#### Correlation between candidate spectral markers and validated EoE activity indices

The spectral ratios at 936/1,449 cm^−1^ related to glycogen content and at 1,301/1,260 cm^−1^ assigned to lipid content significantly decreased with increasing EoEHSS score (*r* = −0.62; *P* = 0.01 and *r* = −0.63; *P* = 0.01, respectively) suggesting that the glycogen content and the lipid content had an inverse relationship with the extent of tissue pathology. There was no significant correlation between spectral intensities assigned to glycogen, proteins, and lipid content total EREFS.

#### Basis for inverse relationship between glycogen and lipid content and tissue pathology

**Figure 5.**
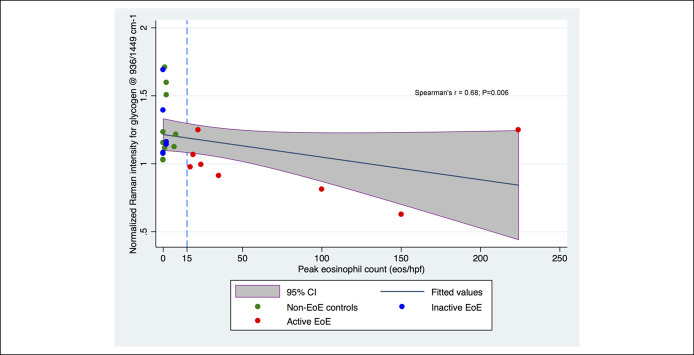
Scatter plot with fitted lines depicting the inverse relationship between glycogen content (at Raman intensity ratio of 936/1,449 cm^−1^) and the peak eosinophil counts by study groups. CI, confidence inerval; EoE, eosinophilic esophagitis.

**Figure 6.**
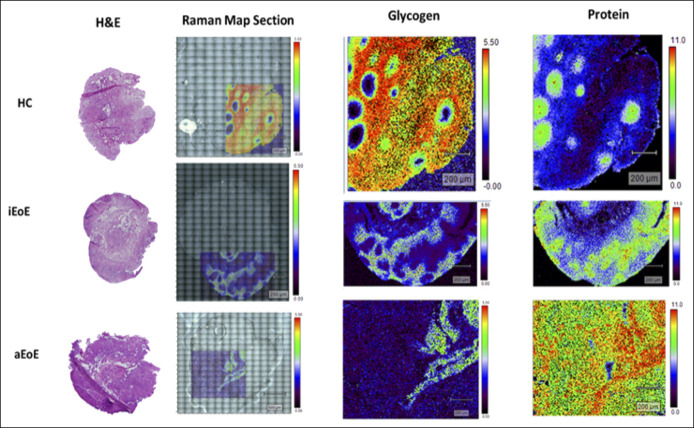
Raman mapping of representative samples from non-EoE controls, inactive EoE and active EoE showing reduced glycogen content in inactive EoE and active EoE compared to non-EoE controls, and abundance of proteins in inactive EoE and active EoE when compared to non-EoE control (magnification 200 μm). EoE, eosinophilic esophagitis.

In this study, we investigated the basis for the inverse relationship between spectral peaks related to glycogen content and lipid content, 936/1,449 cm^−1^ and 1,301/1,260 cm^−1^, respectively, with the extent of tissue pathology per EoEHSS. We found that the inverse relationship for glycogen content was driven by increasing EI (*r* = − 0.53; *P* = 0.05), and this inverse relationship was accentuated when we used PEC as a surrogate of EI and a continuous variable (*r* = −0.68; *P* = 0.006) (Figure 5). An inverse relationship was also noted between glycogen content and BZH (*r* = −0.55; *P* = 0.04) (see Figure 4, Supplementary Digital Content 4, http://links.lww.com/CTG/A303). Similarly, the inverse relationship between the lipid content and tissue pathology was driven by increasing EI (*r* = −0.61; *P* = 0.01) (see Figure 5, Supplementary Digital Content 5, http://links.lww.com/CTG/A304), PEC (*r* = −0.53; *P* = 0.04), and BZH (*r* = −0.63; *P* = 0.01) (see Figure 6, Supplementary Digital Content 6, http://links.lww.com/CTG/A305).

### Raman mapping

Raman mapping allowed a deeper understanding of the spatial distribution of the candidate spectral markers associated with each of the study groups. Spatial maps generated at the Raman bands of glycogen revealed significantly lower intensities of glycogen in aEoE and iEoE samples compared with that in samples of non-EoE controls, with aEoE samples displaying the least amount of glycogen in its epithelium. In addition, maps constructed from Raman bands attributed to protein structure revealed that higher protein content was noted in aEoE samples when compared with those in iEoE samples and samples of non-EoE controls, with a relatively higher protein intensity noted in iEoE samples when compared with that in samples of non-EoE controls (Figure 6).

## DISCUSSION

In this prospective, *in vitro* study, using Raman spectroscopy, we found key differences in the spectral intensities assigned to glycogen, protein, and lipid content in children with EoE compared with non-EoE controls. These differences hold potential to serve as spectral markers of both EoE activity status and the extent of pathology. The findings from our spectral analysis corroborated with Raman mapping performed on an independent set of samples. These novel findings show, for the first time, that the biochemical composition of the esophageal mucosa, as identified by Raman spectroscopy, is altered in EoE, and this might have diagnostic and prognostic implications.

When compared with non-EoE controls, the peak intensities related to glycogen and lipid content decreased in a stepwise manner in iEoE and aEoE states. Furthermore, the peak intensities representing the glycogen and lipid content inversely correlated with the degree of tissue pathology as assessed by EoEHSS. This inverse relationship seemed to be majorly driven by the degree of eosinophilic inflammation (and PEC) and BZH. We hypothesize that a combination of increased glycogen uptake by the large numbers of eosinophils, abundance of immature epithelial cells in basal zone with lower amounts of cytoplasm and cytoplasmic glycogen, and ongoing metabolic activity related to the eosinophilic inflammation (23) in epithelial (and possibly in subepithelial) compartment might explain these findings. The intensity of Raman peaks assigned to lipid content in iEoE was higher than that of aEoE but comparable with that of non-EoE controls. This increase in lipid content in iEoE samples compared with those in aEoE samples and non-EoE controls might be reflective of underlying tissue healing process as previously demonstrated in the *in vivo* analysis of mucosal lipid content in patients with quiescent ulcerative colitis compared with those with active ulcerative colitis (24).

Next, the peak intensities of spectral signatures suggestive of protein content were higher in EoE when compared with those in non-EoE controls. On average, the protein content of iEoE samples is slightly decreased compared with that in aEoE samples, based on the average of only 6–8 point measurements across the tissue, but this difference was not observed to be significant across all patients. However, if we consider the distribution of the protein content for iEoE samples in the Raman map (Figure 6), we can see that there is a higher amount of protein compared with that in non-EoE controls, and in certain areas, this protein amounts to similar intensities to aEoE samples but, in other areas, similar to non-EoE controls. The Raman map also suggests that iEoE has a lower overall protein Raman band intensity than aEoE. The Raman maps provide a larger field of view (1.0–1.4 mm^2^) with a 6-µm step-size compared with average point spectra with a field of 0.5 µm from 6 to 8 dispersed areas of the tissue. Therefore, future studies will focus on assessing a larger field of view to further identify how the change in protein content can be used to differentiate esophageal disease state. This relative increase in protein content in aEoE cases when compared with iEoE cases and non-EoE controls and in iEoE cases when compared with non-EoE controls could be related to the influx of cytokines, chemokines, and inflammatory cells resulting in the microstructural and cellular changes and the characteristic dense eosinophilic inflammation. These hypotheses will need to be tested in future mechanistic studies. Nevertheless, these findings can help distinguish iEoE and aEoE cases and non-EoE controls at the current time.

This study has some limitations. The major limitation is that we did not conduct biochemical assays to quantify glycogen, protein, or lipid content in our samples. Nonetheless, we used Raman peak assignments that are widely accepted in Raman studies. Our sample size was relatively small when compared with some of the studies published in esophageal oncology literature (25,26). However, the prevalence of pediatric EoE is lower when compared with the burden of esophageal premalignant or malignant conditions. Regardless of the sample size (which would tend to bias our findings toward the null), we still demonstrated differences between the study groups and generated hypotheses for future studies. Another drawback is that the Raman spectra were not acquired from the exact same biopsy samples that were scored for tissue pathology. This was not practically feasible because the clinical biopsies were placed in formaldehyde that modifies the biochemical composition of the sample and precludes Raman spectroscopy. To address this issue, we acquired Raman spectra from samples collected from an adjacent site in the esophagus. In addition, this study involved children with inflammatory phenotype of EoE; therefore, the results might not be applicable to adult EoE patients or EoE patients with fibrostenotic phenotype of EoE.

Despite these limitations, our study has several strengths. It was prospective, with rigorous data collection. It was the first study to apply Raman spectroscopy to elucidate biochemical changes associated with EoE. We identified a panel of spectral biomarkers associated with aEoE and iEoE cases and non-EoE controls. A standardized protocol was used to acquire *in vitro* Raman spectra. This allowed us to minimize the possibility of measurement bias and providing a basis for gathering consistent data from future studies. We used validated endoscopic and histologic EoE activity indices. Finally, Raman mapping was performed on an independent set of samples to profile the biochemical and spatial distribution of candidate spectral makers, and the findings from Raman mapping substantiated our results from spectral Raman analysis.

In conclusions, there is growing interest in applying light-based techniques to elucidate the biomolecular, structural, and biochemical changes related to tissue states (healthy or diseased) (27). We used Raman spectroscopy to profile the biochemical changes associated with EoE and identified spectral biomarkers associated with EoE activity status and the extent of tissue pathology. Efforts are underway to validate our findings in a larger cohort of pediatric and adult patients with EoE and with inflammatory and fibrostenotic EoE phenotypes, and with a through-the-scope probe for *in vivo* use. Longitudinal data are also being collected to assess changes during the evolution of disease between active and inactive disease and between disease phenotypes.

## CONFLICTS OF INTEREST

**Guarantor of the article:** Girish Hiremath, MD, MPH.

**Specific author contributions:** Girish Hiremath, MD, MPH, and Andrea Locke, PhD, share co-first authorship. G.H. and A.L. equally contributed in conceptualizing the study, acquisition, analysis, and interpretation of data, drafting the manuscript, critical revision of the manuscript for important intellectual content, and statistical analysis; obtained funding, technical, and material support. G.T. contributed in conceptualizing the study, acquisition, analysis, and interpretation of data, critical revision of the manuscript for important intellectual content, statistical analysis, and technical support. R.G. contributed in analysis and interpretation of data, critical revision of the manuscript for important intellectual content, statistical analysis, and technical support. S.A. contributed in conceptualizing the study, critical revision of the manuscript for important intellectual content, and administrative and material support. H.C. contributed in conceptualizing the study, acquisition, analysis and interpretation of data, and critical revision of the manuscript for important intellectual content. E.S.D. contributed in conceptualizing the study, critical revision of the manuscript for important intellectual content, interpretation of data, and study supervision. A.M.-J. contributed in conceptualizing the study, analysis and interpretation of data, critical revision of the manuscript for important intellectual content, statistical analysis, obtained funding, administrative, technical and material support, and study supervision. Each of the authors has approved the final draft submitted.

**Financial support:** G.H. is supported by the American College of Gastroenterology Junior Faculty Development Award, the Eunice Kennedy Shriver National Institute of Child Health & Human Development of the National Institutes of Health (NIH) under Award Number K12HD087023. A.L. is supported by the Vanderbilt's Academic Pathways Postdoctoral Fellowship. G.T. is supported by R01CA212147 from the NIH (awarded to A.M.-J). R.G. is supported by R01HD081121 from the NIH (awarded to A.M.-J) and Vanderbilt Internal Trans-Institutional Programs (TIPs) award (awarded to A.M.-J). A.M.-J. is supported by R01CA212147 and R01HD081121 from the NIH. The content is solely the responsibility of the authors and does not necessarily represent the official views of the NIH.

**Potential competing interests:** None to report.

**Study Highlights**

**WHAT IS KNOWN**

✓ Despite significant advances, pathogenesis of eosinophilic esophagitis (EoE) remains unclear.
✓ This is partly due to lack of our understanding of the tissue-level biochemical changes associated with EoE.
✓ Raman spectroscopy can be used to profile biochemical alterations associated with tissue-specific states.

**WHAT IS NEW HERE**

✓ This is the first study to apply Raman spectroscopy to profile biochemical changes in the esophageal samples obtained from children with and without EoE.
✓ Spectral traits related to biochemical alterations associated with EoE activity status and the extent of tissue pathology were identified.
✓ Elucidating tissue-level biochemical alterations using Raman spectroscopy can provide novel insights into EoE pathobiology.

**TRANSLATIONAL IMPACT**

✓ Our results provide support for initiation of adequately powered studies to optimize the application of Raman spectroscopy in EoE.
✓ Identifying biomolecular composition can foster identification of novel diagnostic, prognostic, and therapeutic targets in EoE.

## Supplementary Material

SUPPLEMENTARY MATERIAL
