# Supplementary material for: Novel Insights Into Tissue-Specific Biochemical Alterations in Pediatric Eosinophilic Esophagitis Using Raman Spectroscopy
Source: Clin Transl Gastroenterol. 2020 Jul 21;11(7):e00195. doi: 10.14309/ctg.0000000000000195 (PMC7386346; doi:10.14309/ctg.0000000000000195)
Supplement: SUPPLEMENTARY MATERIAL [file ct9-11-e00195-s001.docx]

**Methods:**

**Raman data: Instrumentation:** inVia^TM^ Raman Confocal microspectroscope (Wotton-Under-Edge, UK) includes a 785 nm master oscillator power amplifier laser for excitation, a high efficiency holographic spectrograph, and a peltier-cooled charge coupled detector device. It is known to reliably analyze samples with uneven, curved, or rough surfaces, and without any need for sample preparation.

**Histologic evaluation:** EoEHSS scoring included eosinophilic inflammation (EI), basal zone hyperplasia (BZH), eosinophilic microabscess (EA), eosinophil surface layering (ESL), dilated intercellular spaces (DIS), surface epithelial alterations (SEA), dyskeratotic epithelial cells (DEC), and lamina propria fibrosis (LPF) when lamina propria was present. Each of these features were graded from 0 to 3. The maximum possible grade score for each biopsy was 24. The final score was the ratio of the sum of the assigned scores for each feature evaluated divided by the maximum possible score for that biopsy. If a feature was not evaluated, then the maximum possible score was reduced by 3. For instance, when lamina propria was not present the maximum possible score was reduced from 24 to 21 because 7 instead of 8 features were evaluated.
